# Pooled Analysis of Sleep Outcomes After Treatment With Temperature‐Controlled Radiofrequency in Nasal Airway Obstruction Patients

**DOI:** 10.1002/oto2.70250

**Published:** 2026-05-15

**Authors:** Masayoshi Takashima, Ashwin Ananth, Paul T. Hoff, Paul Schalch Lepe, Gavin Setzen, Maria V. Suurna

**Affiliations:** ^1^ Department of Otolaryngology–Head & Neck Surgery Houston Methodist Hospital Houston Texas USA; ^2^ Eastern Shore ENT Fairhope Alabama USA; ^3^ Department of Otolaryngology–Head and Neck Surgery University of Michigan Medical School Ann Arbor Michigan USA; ^4^ Michigan Otolaryngology Surgery Associates Ypsilanti Michigan USA; ^5^ Silenso Clinic San Diego California USA; ^6^ Departments of Otolaryngology–Head and Neck Surgery and Sleep Medicine UC San Diego Health San Diego California USA; ^7^ Albany ENT and Allergy Services, P.C. Albany New York USA; ^8^ Department of Otolaryngology Albany Medical College Albany New York USA; ^9^ Department of Otolaryngology–Head and Neck Surgery University of Miami Health System Miami Florida USA

**Keywords:** nasal airway obstruction, nasal valve dysfunction, obstructive sleep apnea, septal swell body, sleep‐disordered breathing, temperature‐controlled radiofrequency

## Abstract

**Objective:**

To assess the long‐term impact of temperature‐controlled radiofrequency (TCRF) treatment on sleep‐related symptoms in patients with nasal airway obstruction (NAO), including those with documented obstructive sleep apnea (OSA).

**Study Design:**

Pooled analysis of prospective multicenter studies.

**Setting:**

Seven academic and 25 private otolaryngology sites.

**Methods:**

A pooled analysis of 345 adults across four prospective multicenter studies (PIVOTAL, VATRAC, SWELL, and AERWAY) from 7 academic and 25 private otolaryngology sites was conducted. Participants had severe‐to‐extreme NAO and received a single, office‐based TCRF treatment targeting the nasal valve or septal swell body (SSB). Outcomes included sleep‐related components of the Nasal Obstruction Symptom Evaluation (NOSE) scale, 22‐item Sino‐Nasal Outcome Test (SNOT‐22) sleep domain, and Epworth Sleepiness Scale (ESS). Analyses included stratification by the presence or absence of OSA and linear regression.

**Results:**

TCRF treatment was associated with significant, durable improvements in sleep‐related symptoms through 24 months. At 24 months, the “Trouble Sleeping” subcomponent of the NOSE score improved by −1.9 (95% CI, −2.0 to −1.7) points, a 64% improvement. SNOT‐22 sleep domain scores declined by −9.0 (95% CI, −10.5 to −7.6) points, with more than 80% of participants achieving clinical improvement. Among those with excessive daytime sleepiness (ESS ≥ 10), ESS dropped by −8.3 (95% CI, −9.7 to −6.9), and the prevalence of sleepiness fell from 53.8% to 9.8%. Improvements were consistent across the OSA+ subgroup.

**Conclusion:**

Office‐based TCRF therapy targeting the nasal valve and/or SSB is associated with clinically meaningful, durable improvements in sleep‐related symptoms and daytime somnolence in NAO patients, including a subset of those with documented OSA.

Nasal airway obstruction (NAO) has a well‐recognized role in sleep‐disordered breathing and obstructive sleep apnea (OSA).^1^ Approximately 70% of patients with OSA have clinically significant NAO,^2^ and nearly 60% of OSA patients report nasal symptoms prior to starting continuous positive airway pressure (CPAP) therapy.^3^ Persistent nasal resistance associated with untreated NAO can impair CPAP and oral appliance effectiveness, contributing to reduced adherence and suboptimal treatment outcomes.^4^, ^5^ When untreated, NAO has been linked to long‐term sequelae of OSA, including increased risks of cardiovascular disease, stroke, diabetes, and cancer.^6^, ^7^ Chronic nasal congestion and habitual mouth breathing also increase OSA risk,^8^ with multiple studies reinforcing the role of upper airway resistance in sleep‐disordered breathing.^8^, ^9^, ^10^

Mechanistically, nasal obstruction increases upstream resistance, necessitating compensatory mouth breathing. This shift in breathing pattern generates negative intraluminal pressure downstream, increasing pharyngeal collapsibility and triggering sleep‐disordered breathing events such as apnea and hypopnea.^10^ Dynamic or reversible collapse is particularly disruptive, as fluctuating nasal resistance amplifies negative inspiratory pressure and restricts airway adaptation during sleep, thereby exacerbating OSA pathophysiology.^9^, ^10^, ^11^

Despite interventions such as septoplasty or turbinate reduction, residual NAO is common due to other anatomical and functional contributors such as nasal valve dysfunction (NVD). Weakness or narrowing of the internal nasal valves, including static and dynamic nasal valve collapse, can cause nasal obstruction and have been implicated in sleep‐disordered breathing,^12^ while the septal swell body (SSB) (a fusiform structure located on the nasal septum) may hypertrophy and further increase nasal resistance, particularly at night with a recumbent sleeping position.^13^ These factors underscore the importance of a comprehensive, multilevel evaluation in order to optimize outcomes in patients with sleep‐related NAO.^14^

Recognizing the relationship between NAO and OSA, the American Academy of Otolaryngology–Head and Neck Surgery (AAO‐HNS) emphasizes that nasal surgery, particularly procedures targeting structural obstruction, can improve sleep quality, reduce daytime sleepiness, and enhance CPAP compliance.^15^ Surgical correction, including septoplasty, turbinate reduction, and nasal valve repair, has been shown to lower nasal airway resistance and support better adherence to CPAP and oral appliance therapies.^8^

Temperature‐controlled radiofrequency (TCRF) treatment has emerged as a minimally invasive alternative that remodels tissues in the nasal valve, resulting in decreased nasal resistance, improved airflow, and NAO symptoms.^16^, ^17^, ^18^, ^19^, ^20^, ^21^, ^22^ TCRF delivers precise, temperature‑regulated energy to the submucosal layer of the lateral nasal wall, causing controlled collagen contraction and stimulation of new collagen formation. This remodeling widens the nasal passage and promotes more stable airflow while preserving the integrity of the overlying mucosa. By targeting key anatomic contributors to nasal resistance, TCRF provides a structural approach to NAO to improve nasal airflow. However, its impact on sleep‐related symptoms remains unclear. The objective of this study was to explore the effect of TCRF treatment on the sleep‐related outcomes of patients with NAO before‐and‐after treatment using pooled analyses of existing clinical data.

## Methods

### Pooled Patient Population and Study Design

Baseline and follow‐up data through 24 months from four prospective, multicenter studies were pooled: PIVOTAL (NCT03290300), VATRAC (NCT04549545), SWELL (NCT05099263), and AERWAY (NCT04277507). Data were sourced from Medrio (San Francisco, CA), an electronic database for each study.^20^, ^22^, ^23^, ^24^ Appropriate written informed consent was obtained from all patients, and ethics committee approval was obtained at each participating center as part of the original studies, including Eastern Virginia Medical School IRB; WCG Institutional Review Board (IRB) (20213899); Vanderbilt University IRB (211769), and Rush University Medical Center IRB (21061506‐IRB04).

### Study Eligibility Criteria

Across studies, participants were aged ≥18 years, seeking treatment for symptomatic nasal obstruction who had previously failed or were dissatisfied with medical therapy, such as decongestants, antihistamines, or nasal steroid sprays, and were willing to undergo an office‑based procedure. Participants were required to have severe or extreme NAO, classified by the Nasal Obstruction Symptom Evaluation (NOSE) scale (score of ≥55). Eligible patients had structural contributors to NAO, such as NVD, confirmed by nasal endoscopy and a positive response to temporary mechanical support maneuvers (eg, Cottle or modified Cottle maneuver or Q‐Tip test) or SSB hypertrophy as primary contributors to NAO. Common exclusion criteria included recent nasal or sinus surgery, anatomic conditions warranting alternative surgical repair (eg, severe septal deviation or polyposis), active infection, pregnancy or lactation, or medical comorbidities judged by the investigator to increase procedural risk or impair healing. Details of study‑specific inclusion and exclusion parameters are provided in Supplemental Table S1, available online.

### Assessment of Anatomic Contributors

Nasal examinations, including nasal endoscopy and Cottle or modified Cottle maneuvers, were used to assess NVD at baseline, according to each study's inclusion criteria. SSB hypertrophy was evaluated by endoscopic findings according to the Catalano grading system, which categorizes the degree of hypertrophy based on the visibility of the ipsilateral middle turbinate. Findings were recorded according to the investigator's judgment following standard otolaryngologic practice at each site.

### Procedure

All four studies involved a single‐session bilateral treatment using the VivAer® ARC Stylus (Aerin Medical, Inc.) connected to the Aerin Console generator to deliver TCRF energy. All procedures were performed by trained otolaryngologists in an office setting using local anesthesia. A nasal speculum was used to visualize the treatment area, and the Stylus was applied to the target tissue in accordance with the manufacturer's instructions. The treatment was standardized for each study using the standard treatment parameters of 60°C, 4 W, 18‐second application, and 12‐second cooling.

**Figure 1:**
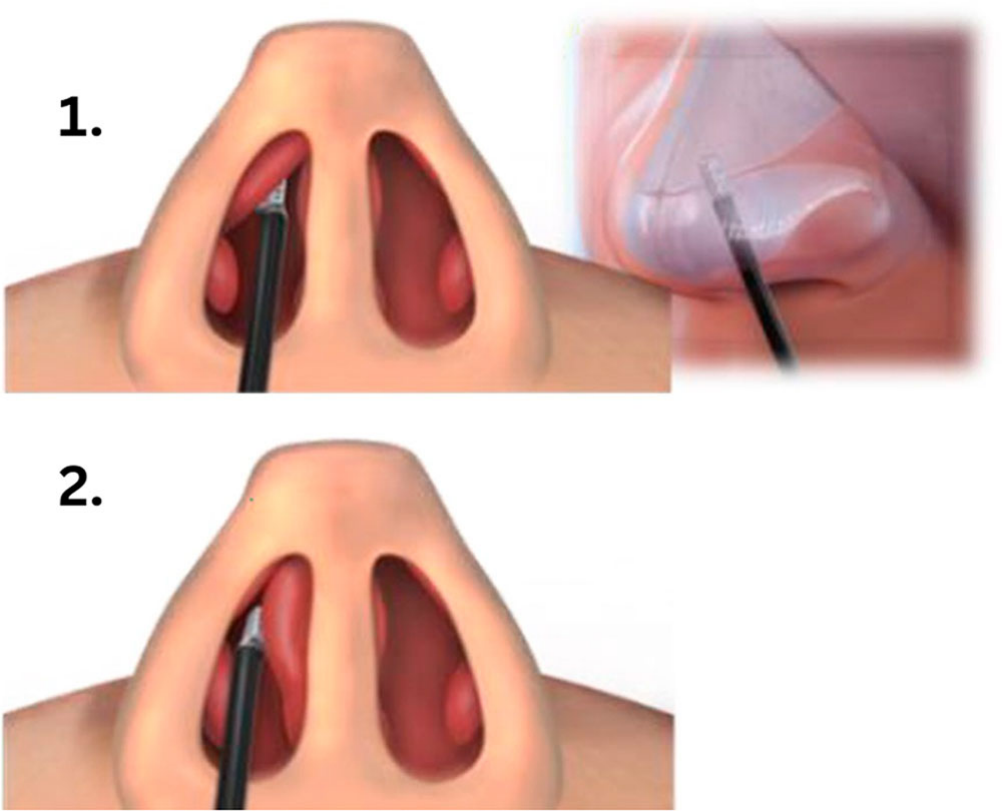
VivAer treatment for nasal airway obstruction. Illustration of intranasal temperature‐controlled radiofrequency (TCRF) treatment targets using the VivAer device. (1) Two views of application to the lateral nasal wall to remodel and stiffen the internal nasal valve, reducing dynamic collapse and increasing airflow. (2) Application to the septal swell body to reduce mucosal bulk and enhance central nasal airflow.

The treatment locations for the PIVOTAL, VATRAC, and AERWAY studies targeted the lateral nasal wall, treating three to four non‐overlapping areas at the junction of the upper and lower lateral cartilage (Figure 1). In contrast, the SWELL study focused on the SSB allowing two to six non‐overlapping applications per nostril. No repeat or touch‐up procedures were permitted nor were inferior turbinates treated in any study.

### Outcome Measures

Sleep‐related outcomes were assessed using the following patient‐reported outcome measures: the NOSE scale, specifically the “Trouble Sleeping” item (Q4), measures the perceived impact of nasal obstruction on sleep continuity and comfort. The question asked: “over the past month, how much of a problem was the following.” Responses were rated using a 5‐point scale (0 = not a problem to 4 = severe problem).^25^, ^26^ The sleep domain of the 22‐item Sino‐Nasal Outcome Test (SNOT‐22) assesses the broader sinonasal quality of life and sleep disturbance associated with nasal and sinus disease. It consists of the following five questions: (Q11) difficulty falling asleep, (Q12) waking at night, (Q13) lack of restful sleep, (Q14) waking up tired, and (Q15) fatigue, and responses were rated using a 6‐point scale (0 = no problem to 5 = as bad as it can be).^27^ Finally, the Epworth Sleepiness Scale (ESS) evaluated subjective daytime sleepiness and functional impairment across common daily situations. It consisted of eight questions that assessed the likelihood of dozing off or falling asleep during daily activities. Responses were rated using a 4‐point scale (0 = no chance to 3 = high chance of falling asleep in described situations).^28^ Together, these instruments capture distinct but complementary clinical domains related to nocturnal nasal obstruction, sleep quality, and daytime alertness.

Lower scores on these instruments reflect reduced sleep‐related symptom burden. For the ESS, a more liberal threshold of ≥10^29^, ^30^ was used as a clinically relevant score indicative of excessive daytime sleepiness (EDS); reductions in score reflect improvement in daytime somnolence. In addition to the ESS cutoff for abnormal daytime sleepiness, a more conservative threshold (ESS ≥ 15) was used to identify participants with severe EDS subtypes, consistent with recent exclusion criteria used for a large, randomized trial evaluating CPAP and cardiovascular risk.^31^ Although there are no formally established responder thresholds for the SNOT‐22 sleep domain, the minimal clinically important difference (MCID) has been defined as a decrease in the composite sleep score of ≥2.9 points, as proposed by Chowdhury et al.^32^

Baseline nasal and sleep‐related comorbidities were assessed across participants in the VATRAC, SWELL, and AERWAY studies using standardized clinical evaluations and patient history. Variables included sleep disorders treatments, presence and laterality of nasal valve collapse, septal deviation (including anatomical location), OSA diagnosis, chronic rhinitis subtype, and history of sinus disease. Nasal valve assessment included differentiation between static, dynamic, and mixed collapse on each side. Diagnoses of rhinitis and sinus disease were recorded based on investigator documentation at study enrollment. These assessments allowed for cross‐cohort comparison of structural and symptomatic contributors to NAO.

### Statistical Analyses

The primary analysis used linear mixed‐effects models (LMMs) with Dunnett‐Hsu adjustments for multiple comparisons versus baseline. Each model included timepoint (visit) as a fixed effect and subject ID as a random intercept to account for repeated measures. The baseline value of the outcome measure (NOSE, SNOT‐22, and ESS) was included as a covariate to adjust for baseline variability and improve the precision of follow‐up comparisons. From these models, adjusted least‐squares (LS) means and 95% confidence intervals were estimated. LS means represent the model‐based marginal mean outcome at each follow‐up timepoint, adjusted for baseline score. Confidence intervals and *P*‐values for changes from baseline were derived from the model using Dunnett‐Hsu adjusted contrasts. Statistical significance was set at *α* = .05. Analyses were conducted on observed data without imputation. The LMM framework accommodated missing observations under the assumption that data were missing at random (MAR). Patients were stratified into two cohorts based on the presence (OSA+) or absence (OSA−) of OSA based on documentation of a previous clinical diagnosis of OSA, when such data were available. Cross‐sectional subgroup comparisons between OSA+ and OSA− cohorts at each visit were evaluated using Welch's *t* tests, which are robust to unequal sample sizes and variances. These subgroup analyses were considered exploratory and were interpreted descriptively. Multiplicity adjustments were applied for repeated measures over time within each outcome, but not across different outcome instruments (NOSE, SNOT‐22, and ESS). All analyses were performed using R Statistical Software (v4.4.1; R Core Team, 2024‐06‐14).

## Results

### Subject Enrollment and Disposition

**Figure 2:**
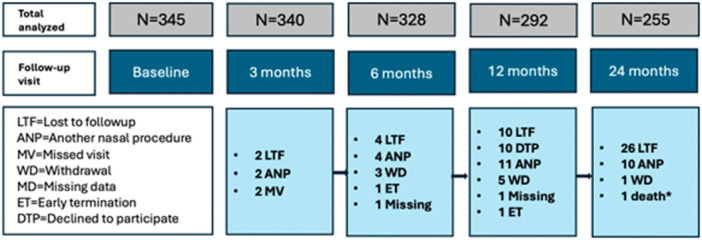
Patient disposition through 24 months. Disposition at baseline and 3 to 24 months across AERWAY, VATRAC, SWELL, and PIVOTAL temperature‐controlled radiofrequency (TCRF) cohorts. Exclusions: Nasal Obstruction Symptom Evaluation (NOSE) < 55 or missing baseline. SWELL late enrollees not study exits. *Death unrelated to device/procedure.

A pooled analysis was conducted using data from 345 patients (PIVOTAL n = 47, VATRAC n = 106, SWELL n = 70, and AERWAY n = 122) across 7 academic and 25 private otolaryngology sites.^20^, ^22^, ^23^, ^24^ Patient disposition and withdrawals were assessed across pooled studies, with reasons for study attrition (eg, loss to follow‐up, voluntary withdrawal, additional nasal procedures, or unrelated death) recorded at each timepoint (Figure 2).^23^, ^25^, ^26^, ^27^

### Demographics and Clinical Characteristics

Demographic characteristics for the pooled cohort (N = 345) are summarized in Table 1. The mean age was 49.1 years (SD: 14.2), with participants ranging in age from 18.9 to 83.7 years. Most participants (75.4%) were under 60 years of age with slightly more males (52.8%). Most participants identified as White (89.6%), with smaller proportions identifying as Hispanic or Latino (7.0%), Asian (2.3%), black or African American (3.8%), American Indian or Alaska Native (0.9%), or not reported (2.5%). Across the studies for which baseline sleep conditions were available, documented OSA status was captured in VATRAC (n = 21) and SWELL (n = 16), accounting for ~11% of participants.

**Table 1:** Demographics

Variable statistic/category	Pooled (N = 345)
Age, y	
Mean (SD)	49.1 (14.2)
Median (min, max)	49.0 (18.9, 83.7)
Age category	
22 to 59 y, n (%)	260 (75.4%)
≥60 y, n (%)	85 (24.6%)
Sex	
Male, n (%)	182 (52.8%)
Female, n (%)	163 (47.3%)
Ethnicity	
Declined to answer	5 (1.5%)
Hispanic or Latino, n (%)	24 (7.0%)
Not Hispanic or Latino, n (%)	316 (91.6%)
Race	
2 or more	3 (0.9%)
American Indian or Alaska Native	3 (0.9%)
Asian	8 (2.3%)
Black or African American	13 (3.8%)
Not reported	9 (2.6%)
White	309 (89.6%)

Abbreviations: n, number of observations; N, number of subjects; SD, standard deviation.

### Change From Baseline in the NOSE Score “Trouble Sleeping” Subcomponent

**Figure 3:**
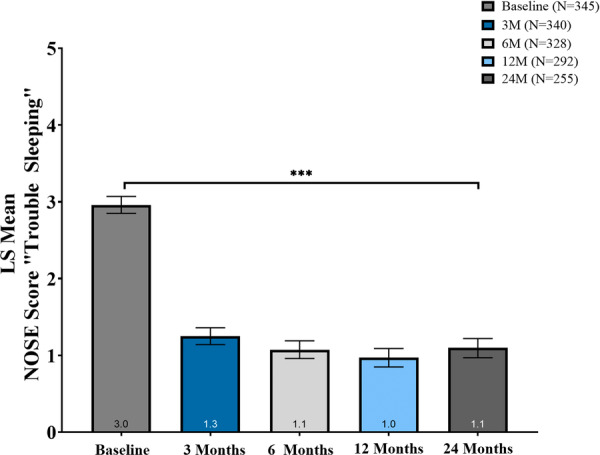
Adjusted mean change in Nasal Obstruction Symptom Evaluation (NOSE) “Trouble Sleeping” score. Least‐squares (LS) mean NOSE Q4 scores declined significantly versus baseline at 3 to 24 months. Error bars: 95% CI from linear mixed‐effects model (LMM). ***P < .001.

The “Trouble Sleeping” subcomponent of the NOSE scale score significantly decreased from a baseline mean score of 3.0 (95% CI, 2.9 to 3.1) at all follow‐up timepoints, with sustained improvement through 24 months (Table 2 and Figure 3). At 3, 6, 12, and 24 months, adjusted LS mean changes from baseline were −1.7 (95% CI, −1.8 to −1.6), −1.9 (95% CI, −2.0 to −1.7), −2.0 (95% CI, −2.1 to −1.8), and −1.9 (95% CI, −2.0 to −1.7), respectively (all *P* < .001). These changes corresponded to mean percent reductions of 57.4%, 62.8%, 66.3%, and 64.1%.

**Table 2:** Change in Nasal Obstruction Symptom Evaluation (NOSE) Q4 “Trouble Sleeping” Subcomponent Scores Through 24 Months^a^

Measure	Baseline (N = 345)	3 mo (N = 340)	6 mo (N = 328)	12 mo (N = 292)	24 mo (N = 255)
*NOSE Q4 “Trouble Sleeping”*					
Severity score					
0 = no symptoms	7 (2.0)	131 (38.5)	142 (43.3)	142 (48.5)	115 (44.7)
1 = mild symptoms	23 (6.7)	75 (22.1)	76 (23.2)	67 (22.9)	58 (22.6)
2 = moderate symptoms	67 (19.4)	67 (19.7)	64 (19.5)	55 (18.8)	50 (19.5)
3 = severe symptoms	127 (36.8)	51 (15.0)	36 (11.0)	18 (6.1)	22 (8.6)
4 = extreme symptoms	121 (35.1)	16 (4.7)	10 (3.0)	11 (3.8)	12 (4.7)
Subcomponent score					
Mean (SD)	3.0 (1.0)	1.3 (1.2)	1.1 (1.2)	0.9 (1.1)	1.0 (1.2)
Median	3.0	1.0	1.0	1.0	1.0
Min‐max	0.0‐4.0	0.0‐4.0	0.0‐4.0	0.0‐4.0	0.0‐4.0
95% CI	(2.9, 3.1)	(1.1, 1.4)	(0.9, 1.2)	(0.8, 1.1)	(0.9, 1.2)
LS mean (95% CI)—Trouble Sleeping (LMM‐adjusted)	2.96 (2.85, 3.07)	1.25 (1.14, 1.36)	1.08 (0.96, 1.19)	0.97 (0.85, 1.09)	1.10 (0.97, 1.22)
CFB					
Mean (SD)	0.0 (0.0)	−1.7 (1.3)	−1.9 (1.3)	−2.0 (1.3)	−1.9 (1.4)
Median	0.0	−2.0	−2.0	−2.0	−2.0
Min‐max	0.0‐0.0	−4.0 to 2.0	−4.0 to 2.0	−4.0 to 3.0	−4.0 to 3.0
95% CI	(0.0, 0.0)	(−1.8, −1.6)	(−2.0, −1.7)	(−2.1, −1.8)	(−2.0, −1.7)
*P*‐value—CFB versus baseline (LMM)	N/A	<.001	<.001	<.001	<.001
LS mean (95% CI) (LMM‐adjusted)	N/A	−1.70 (−1.82, −1.58)	−1.87 (−1.99, −1.75)	−1.96 (−2.09, −1.84)	−1.83 (−1.97, −1.69)
% CFB					
Mean (SD)	0.0 (0.0)	−57.4 (45.2)	−62.8 (44.7)	−66.3 (45.4)	−64.1 (42.8)
Median	0.0	−66.7	−75.0	−75.0	−75.0
Min‐max	0.0‐0.0	−100.0 to 200.0	−100.0 to 200.0	−100.0 to 300.0	−100.0 to 200.0
95% CI	(0.0, 0.0)	(−62.3, −52.6)	(−67.7, −57.9)	(−71.4, −60.9)	(−69.5, −58.8)

Abbreviations: CFB, change from baseline; CI, confidence interval; LMM, linear mixed model with visit as fixed effect, baseline score as covariate, and subject ID as random intercept; LS, least‐squares; SD, standard deviation.

^a^ Proportions of patients reporting each severity level (0 = no symptoms to 4 = extreme symptoms) for the NOSE Q4 “Trouble Sleeping” subcomponent are shown over time for the pooled cohort (N = 345 at baseline). Percentages are based on non‐missing responses at each visit. Negative values denote improvement. Percent change values (%CFB) reflect proportional change relative to baseline. Change = result − baseline; % change = (change/baseline) × 100. LS means are adjusted for baseline scores. *P*‐values are derived from LMM analysis with Dunnett‐Hsu adjustment for multiple comparisons versus baseline, not *t* tests.

### Severity of the NOSE Scale Score “Trouble Sleeping” Subcomponent by Visit

**Figure 4:**
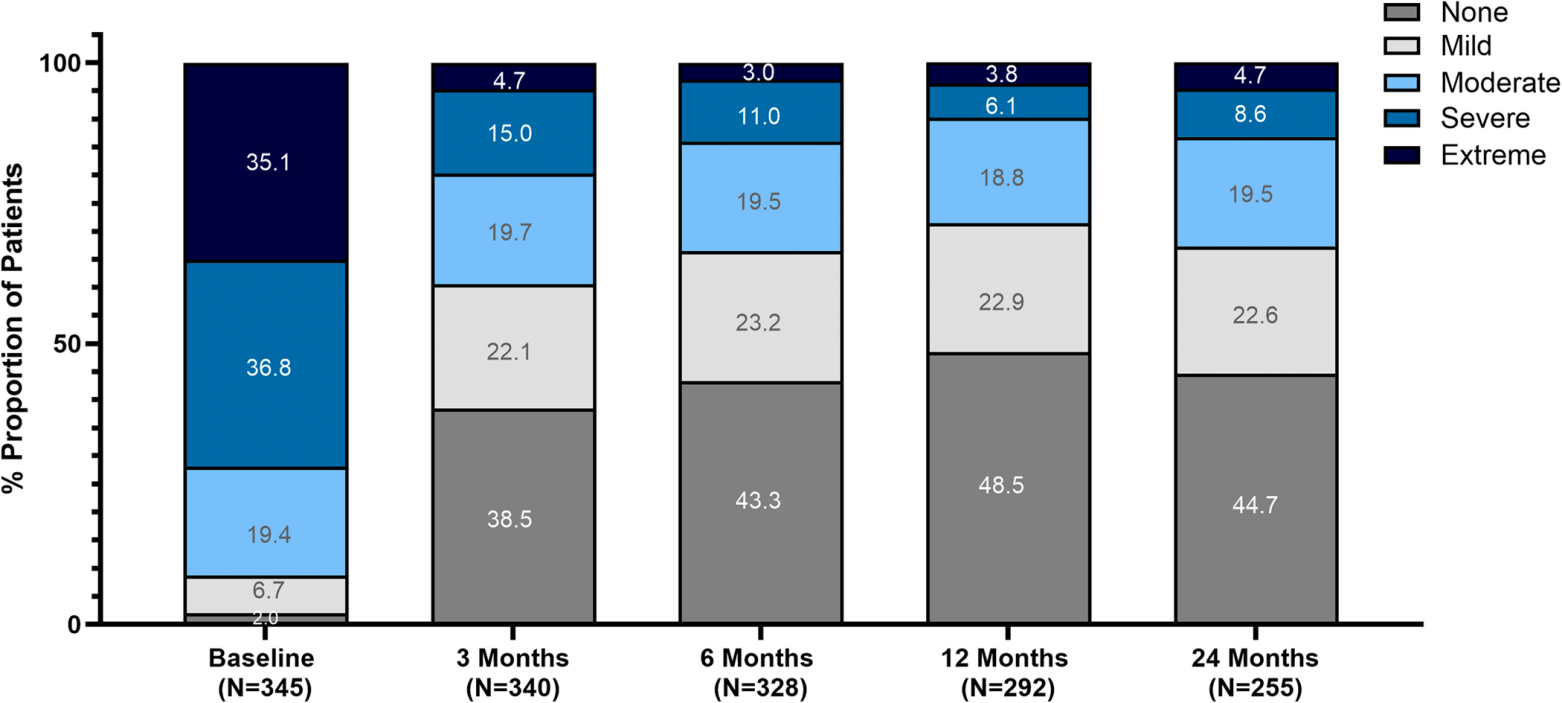
Severity distribution of Nasal Obstruction Symptom Evaluation (NOSE) Q4 “Trouble Sleeping”. Stacked bars show NOSE Q4 severity categories at baseline and 3 to 24 months after temperature‐controlled radiofrequency (TCRF) treatment.

Reported severity of sleep‐related nasal obstruction, as measured by the “Trouble Sleeping” subcomponent of the NOSE score, showed a pronounced and sustained shift toward lower symptom burden following TCRF treatment (Figure 4). At baseline, nearly three‐quarters of participants (71.9%) rated their symptoms as severe or extreme (score of 3 or 4). This proportion declined sharply by 3 months (19.7%) and remained low through 12 and 24 months (9.9% and 13.3%, respectively). These trends reflect a durable, clinically meaningful reduction in sleep‐related nasal obstruction symptoms for most treated individuals.

### Impact of OSA Status on NOSE Score Improvement

The “Trouble Sleeping” component of the NOSE score improved significantly over time in OSA+ and OSA− (including true negatives and unknown) participants. At baseline, the adjusted LS mean “Trouble Sleeping” score was 3.1 (95% CI: 2.8 to 3.4) in the OSA+ group and 2.9 (95% CI, 2.8 to 3.0) in the OSA− group (Supplemental Table S2, available online). At 3 months, both groups showed lower scores compared with baseline; however, the between‑group *P*‑value (*P* = .003) indicated a temporary difference in absolute scores, with OSA+ participants reporting slightly higher residual symptom scores than OSA− participants. By 6 months and through 24 months, no substantial differences were observed between groups, demonstrating that TCRF treatment was associated with sustained and comparable improvement across patients with and without documented OSA.

### SNOT‐22 Sleep Domain

**Figure 5:**
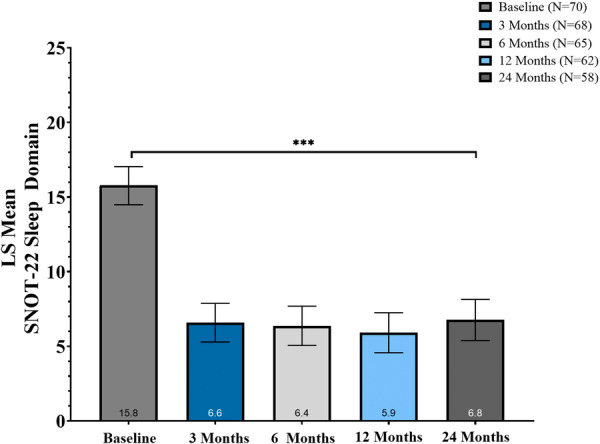
Change in 22‐item Sino‐Nasal Outcome Test (SNOT‐22) sleep domain through 24 months. Adjusted least‐squares (LS) mean SNOT‐22 sleep scores fell significantly at all visits (***P < .001) after temperature‐controlled radiofrequency (TCRF) treatment. Error bars: 95% CI from linear mixed‐effects model (LMM).

In the SWELL study, sleep‐related quality of life, as measured by the SNOT‐22 sleep domain, improved significantly from baseline through 24 months (Table 3). The mean baseline SNOT‐22 sleep domain score was 15.8 (95% CI, 14.5 to 17.0), decreasing to 6.6 at 3 months and remaining stable through 24 months (6.7 at 24 months). The adjusted LS mean change from baseline ranged from −9.2 (95% CI, −10.6 to −7.8) at 3 months to −9.0 (95% CI, −10.5 to −7.6) at 24 months, with all changes statistically significant (*P* < .001) (Figure 5). Percent reductions from baseline averaged 57.6% to 61.8% across follow‐up visits, reflecting substantial and sustained improvements in sleep‐related symptom burden.

**Table 3:** Longitudinal Changes in 22‐Item Sino‐Nasal Outcome Test (SNOT‐22) Sleep Domain Scores Over 24 Months in the SWELL Cohort^a^

Measure	Baseline (N = 70)	3 mo (N = 68)	6 mo (N = 65)	12 mo (N = 62)	24 mo (N = 58)
*Longitudinal changes in SNOT‐22 sleep domain scores*					
Mean (SD)	15.8 (6.1)	6.6 (6.1)	6.4 (6.4)	6.0 (5.9)	6.7 (6.5)
Median	16.0	5.5	5.0	4.0	5.0
Min‐max	0.0‐25.0	0.0‐20.0	0.0‐24.0	0.0‐23.0	0.0‐22.0
95% CI	(14.3, 17.2)	(5.2, 8.1)	(4.9, 7.9)	(4.5, 7.4)	(5.0, 8.4)
LS mean (95% CI)—total score (LMM‐adjusted)	15.8 (14.5, 17.0)	6.6 (5.3, 7.9)	6.4 (5.1, 7.7)	5.9 (4.6, 7.3)	6.8 (5.4, 8.1)
*CFB*					
Mean (SD)	0.0 (0.0)	−9.2 (6.8)	−9.4 (6.4)	−9.9 (7.5)	−8.8 (7.8)
Median	0.0	−8.0	−9.0	−9.0	−8.0
Min‐max	0.0‐0.0	−24.0 to 3.0	−22.0 to 3.0	−25.0 to 7.0	−25.0 to 7.0
95% CI	(0.0, 0.0)	(−10.8, −7.6)	(−11.0, −7.9)	(−11.8, −8.1)	(−10.8, −6.8)
LS mean (95% CI) (LMM‐adjusted)	N/A	−9.2 (−10.6, −7.8)	−9.4 (−10.8, −7.9)	−9.8 (−11.3, −8.37)	−9.0 (−10.5, −7.6)
*P*‐value—CFB value compared to baseline (LMM)	N/A	<.001	<.001	<.001	<.001
*% CFB*					
Mean (SD)	0.0 (0.0)	−57.6 (35.8)	−61.8 (35.1)	−60.6 (35.8)	−54.9 (44.0)
Median	0.0	−57.1	−66.7	−60.0	−63.6
Min‐max	0.0‐0.0	−100.0 to 42.9	−100.0 to 23.1	−100.0 to 43.8	−100.0 to 100.0
95% CI	(0.0, 0.0)	(−66.2, −49.1)	(−70.4, −53.2)	(−69.6, −51.6)	(−66.3, −43.5)
*MCID*					
n (%)	N/A	56 (82.4%)	53 (81.5%)	52 (83.9%)	47 (81.0%)
95% CI	N/A	(71.6%, 89.6%)	(70.4%, 89.1%)	(72.8%, 91.0%)	(69.1%, 89.1%)

Abbreviations: %CFB, percent change from baseline; CFB, change from baseline; CI, confidence interval; LMM, linear mixed‐effects model; LS, least‐squares; MCID, minimal clinically important difference; n, number of observations; N, number of subjects; OSA, obstructive sleep apnea; SD, standard deviation.

^a^ Longitudinal changes were analyzed using LMMs with visit as a fixed effect, baseline score as a covariate, and subject ID as a random intercept. LS means and 95% confidence intervals are derived from the LMM, with Dunnett‐Hsu multiplicity adjustment for comparisons versus baseline. Percent changes are descriptive; statistical testing was based on the LMM. The MCID for the SNOT‐22 sleep domain was defined as an improvement of ≥2.9 points.

Across all follow‐up timepoints, more than 80% of participants achieved the MCID of ≥2.9 points in the SNOT‐22 sleep domain. Specifically, 82.4% achieved MCID in the sleep domain at 3 months, with similarly high rates at 24 months (81.0%). These results indicate the clinical relevance and durability of treatment effects on sleep parameters in patients with sinonasal disease.

### Epworth Sleepiness Scale Scores

**Figure 6:**
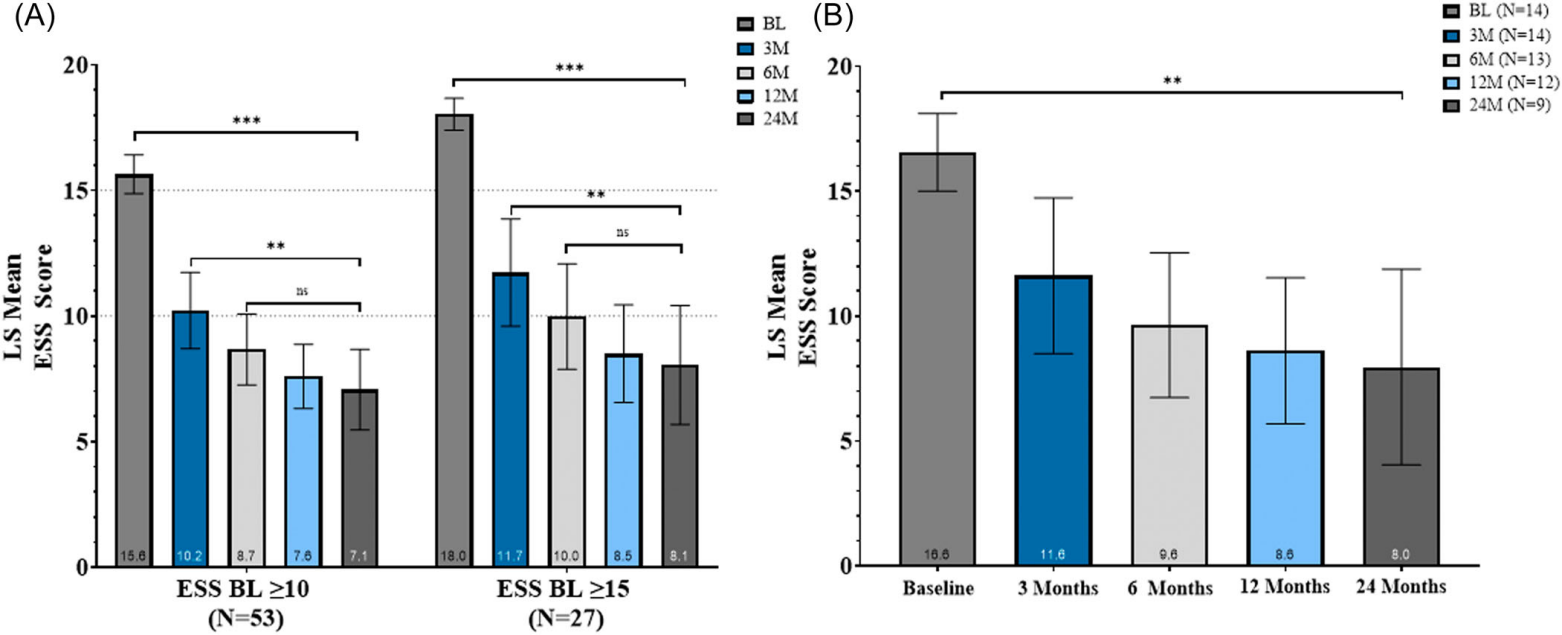
Epworth Sleepiness Scale (ESS) improvements. (A) Least‐squares (LS) mean ESS decreased in VATRAC participants with baseline ESS ≥ 10 or ≥ 15. (B) Obstructive sleep apnea (OSA)+ subgroup with ESS ≥ 10 also improved. Error bars: 95% CI from linear mixed‐effects model (LMM).

Daytime sleepiness, as assessed by the ESS, demonstrated substantial and sustained improvement over 24 months in the VATRAC study (Figure 6A). For patients with ESS scores ≥ 10 (n = 53), adjusted LS mean scores decreased from 15.6 (95% CI, 14.5 to 16.8) at baseline to 7.6 (95% CI, 6.4 to 8.8) at 12 months and 7.1 (95% CI, 5.8 to 8.4) at 24 months. Statistically significant improvements in ESS scores were observed at all follow‐up timepoints compared with baseline (all *P* < .001). The adjusted LS mean change from baseline ranged from −5.4 (95% CI, −6.8 to −4.11) to −8.3 (95% CI, −9.7 to −6.9), with reductions up to 57.6% at 24 months. Participants with more severe daytime sleepiness (ESS ≥  15; n = 27) experienced even greater absolute improvements. Adjusted LS mean ESS scores ranged from 18.0 (95% CI, 16.4 to 19.7) at baseline to 8.1 (95% CI, 6.1 to 10.0) at 24 months (Supplemental Table S3, available online). Adjusted LS mean changes from baseline ranged from −6.3 to −9.7 points, with a 58.5% mean reduction at 2 years.

A similar trend was also observed in the ESS scores in patients who had documented OSA+ and baseline ESS scores ≥ 10 (Figure 6B). These data suggest clinically meaningful symptom relief for individuals with excessive‐to‐severe daytime sleepiness at baseline. Changes in categorical severity levels further underscore the impact of TCRF treatment. The proportion of participants with ESS scores above the clinical threshold (ESS ≥ 10) fell from 53.8% at baseline to 9.8% at 24 months. Meanwhile, those with scores in the normal range (ESS ≤ 9) increased from 46.3% to 90.3%, representing a major and durable shift toward typical sleepiness levels post‐treatment. Furthermore, an exploratory LMM‐based pairwise analysis comparing pairwise successive study visits (3 vs 6 months, 6 vs 12 months, and 12 vs 24 months) found improvements between the 3‐ versus 6‐month timepoint (*P* = .014) with no changes thereafter (Supplemental Table S3, available online).

## Discussion

The link between sleep and overall health is well established. A recent meta‐analysis of 65 randomized controlled trials involving more than 8600 participants found that sleep‐improving interventions such as cognitive behavioral therapy for insomnia, image rehearsal therapy, sleep education, and acupuncture led to reductions in depression, anxiety, stress, and psychosis symptoms.^33^ These findings highlight the impact of improving sleep on mental health and highlight the need to identify and treat physiological contributors to poor sleep.

Despite its clinical burden, many patients with NAO are untreated or managed pharmacologically, even with the availability of effective interventions for NVD. Factors such as NVD or SSB hypertrophy may go unrecognized in patients presenting with fatigue, insomnia, or nonrestorative sleep. Surgical interventions, including septoplasty, turbinate reduction, and functional rhinoplasty, are available but carry risks (eg, bleeding, infection, and septal perforation) and a higher cost, which may reduce the appeal of surgery.^34^, ^35^, ^36^, ^37^ Office‐based interventions such as J‐flaps and absorbable implants (eg, Latera) can improve nasal valve stability with relatively low procedural risk; however, published outcomes beyond 12 months are limited.^38^, ^39^, ^40^ These considerations highlight a therapeutic gap for patients with NAO and sleep disturbances, one that may be addressed by TCRF.

In this study, the “Trouble Sleeping” component of the NOSE score demonstrated rapid and sustained improvements through 24 months, with mean reductions exceeding 57% from baseline, underscoring the long‐term benefits on sleep‐related impairments. These improvements were observed in the overall NAO population and were consistent among the small subgroup of participants with documented OSA, who demonstrated similar directional changes. SNOT‐22 sleep domain scores also declined significantly and remained improved over time. ESS data from VATRAC revealed more modest but clinically relevant trends. Among participants with baseline scores ≥10, mean scores declined significantly through 24 months. The proportion of individuals above the excessive daytime sleepiness threshold (ESS ≥ 15), a level that has been associated with sleep disorders linked to increased cardiovascular risk,^31^ decreased from 25.5% to 4.2%, suggesting a potential long‐term benefit in reducing sleep‐related contributors in patients with cardiovascular morbidity. A longitudinal analysis assessing visit‐to‐visit changes also demonstrated continued improvements in sleep beyond 3 months, indicative that sleep improvement evolves over time. The findings demonstrate that targeted treatment of NVD or SSB can lead to meaningful and sustained improvements in both nasal obstruction and sleep‐related symptoms.

The mechanism underlying these improvements may extend beyond subjective symptom relief. Nasal obstruction increases upstream resistance, often leading to compensatory mouth breathing. According to the Starling resistor model,^11^ this shift promotes negative intraluminal pressure downstream, increasing pharyngeal collapsibility and triggering sleep‐disordered breathing events.^10^ Catalano et al validated this model, demonstrating that treating nasal obstruction led to significant increases in retrolingual space volume and minimum pharyngeal cross‐sectional areas in adults and children, without any pharyngeal or nasopharyngeal intervention.^41^ By improving airflow through the nasal valve or SSB, TCRF may reduce collapsibility of the downstream airway, offering a physiologic explanation for the observed sleep benefits.

While this pooled analysis provides valuable insights, several limitations warrant consideration. First, each of the study designs varied between studies (ie, SNOT‐22 was captured only in SWELL and ESS only in VATRAC). This heterogeneity led to missing data for several assessments at different timepoints. While imputation for missing data could have been conducted, it could further introduce bias and underestimate the true variability and standard errors of the estimated parameters. Second, the attrition rate was relatively high (26%), though within the expected range for long‐term studies,^42^ and patients with unfavorable outcomes may have been underrepresented. Third, OSA status was collected as a historical diagnosis, and information regarding whether those participants were using CPAP or other OSA treatments during the study period was not available; therefore, contributions of concurrent therapy to outcomes cannot be ruled out. Consequently, objective sleep parameters such as the apnea‑hypopnea index (AHI) were not collected, as the original trials were only designed to evaluate outcomes related to NAO. However, the available data mirror those observed in participants without documented OSA, suggesting that the parallel reductions in ESS and NOSE scores among participants with OSA may reflect symptom improvements comparable to those without OSA. Furthermore, this analysis was not designed or powered to evaluate objective OSA‑specific outcomes; rather, the results reflect improvements in sleep‑related symptoms in patients with NAO and comorbid OSA, not objective measures of OSA treatment efficacy. Finally, the studies were conducted as nonrandomized, single‐arm trials (with the exception of the VATRAC trial), relying on subjective patient‐reported outcomes, which introduces the potential placebo effect. Nonetheless, improvements in the “Trouble Sleeping” item are consistent with those published by Silvers et al (Δ−1.8 points) compared with sham (Δ−0.7 points) suggesting that the observed benefit exceeds that of placebo.^43^ Moreover, objective data from Pritikin et al^19^ found significant anatomical reductions in SSB thickness on CT imaging following TCRF treatment, further supporting a physiologic rather than placebo‐driven mechanism of action.

Despite these limitations, the analysis possesses several notable strengths. It represents the largest interventional cohort to date, drawn from multiple institutions, which allowed for robust multivariable analyses to identify patient‐specific treatment outcomes. The consistent, significant improvements observed across various measures, including the sleep component of the NOSE score, the SNOT‐22 sleep domain, and ESS, reinforce the clinical impact of TCRF treatment on sleep. Moreover, the before‐and‐after design employing validated patient‐reported instruments, as well as the alignment of these findings with those from a previous randomized controlled trial, bolsters the reliability and generalizability of the results. Finally, the evidence captured in these studies underscores the long‐term beneficial impact that TCRF treatment has on improving sleep outcomes in patients with NAO.

## Conclusion

This pooled analysis suggests that a minimally invasive TCRF procedure targeting the nasal valve and SSB is associated with clinically meaningful and durable improvements in sleep‐related symptoms and daytime sleepiness among patients with NAO.

## Authors' Note

The sponsor provided access to study data and statistical programming support. The authors had full control over analysis specifications, data interpretation, and the decision to submit the manuscript.

Off‐label or investigational use: Not applicable.

Clinical trial: The manuscript is a pooled study of data from four clinical trials: PIVOTAL (https://clinicaltrials.gov/ NCT03290300), VATRAC (https://clinicaltrials.gov/ NCT04549545), SWELL (https://clinicaltrials.gov/ NCT05099263), and AERWAY (https://clinicaltrials.gov/ NCT04277507).

## Author Contributions

**Masayoshi Takashima**, conceptualization, writing—review and editing; **Ashwin Ananth**, conceptualization, writing—review and editing; **Paul T. Hoff**, conceptualization, writing—review and editing; **Paul Schalch Lepe**, conceptualization, writing—review and editing; **Gavin Setzen**, conceptualization, writing—review and editing; **Maria V. Suurna**, conceptualization, writing—review and editing.

## Disclosures

### Competing interests

Dr. Masayoshi Takashima is a consultant to Aerin Medical, Medtronic, and Spirair. Dr. Gavin Setzen is a consultant to Aerin Medical, Stryker ENT, and Cryosa Inc. Dr. Paul Schalch Lepe is a consultant to Aerin Medical and LivaNova. Dr. Paul Hoff is Senior Medical Director to Inspire Medical Systems and Safety Monitor to Cryosa Inc. Dr. Maria V. Suurna is a consultant to Inspire Medical and Nyxoah SA. Dr. Ashwin Ananth is a consultant to Aerin Medical.

### Funding source

The sponsor provided access to the de‐identified study data sets and statistical support for the pooled analyses. No author received direct compensation for manuscript writing.

## Supporting information

Supporting Information.
