# Supplementary material for: Pooled Analysis of Sleep Outcomes After Treatment With Temperature‐Controlled Radiofrequency in Nasal Airway Obstruction Patients
Source: OTO Open. 2026 May 15;10(2):e70250. doi: 10.1002/oto2.70250 (PMC13179042; doi:10.1002/oto2.70250)
Supplement: Supplementary file 1 — Supporting Information. [file OTO2-10-e70250-s001.docx]

**Supplementary Materials**

**Supplementary Table 1. Study Inclusion and Exclusion Criteria**

| **VATRAC** | **SWELL** | **AERWAY** | **PIVOTAL** |
| --- | --- | --- | --- |
| **Inclusion Criteria** | | | |
| Age 18 to 85 years (inclusive) | Age 22 to 85 years (inclusive) | Age 18 or older | 22 Years to 75 Years (inclusive) |
| Seeking treatment for nasal obstruction and willing to undergo an office-based procedure. | Seeking treatment for nasal obstruction and willing to undergo an office-based procedure | Seeking treatment for nasal obstruction. | Seeking treatment for nasal obstruction and willing to undergo an office-based procedure |
| Baseline NOSE score ≥ 55. | Baseline NOSE score ≥ 55 | Baseline NOSE score ≥ 60 | Baseline NOSE score ≥ 60 |
|  | Presence of SSB hypertrophy limiting visualization of the middle turbinate by more than 50%. |  |  |
|  | Reduction in size of the septal swell body (SSB) after application of topical decongestant on a cotton plug directly to the SSB region. |  |  |
|  | Improvement in the symptoms of nasal obstruction after SSB decongestion suggesting that the SSB may play a role in nasal obstruction. |  |  |
| Nasal valve is a primary or significant contributor to the subject's nasal obstruction as determined by the study investigator (based on clinical presentation, physical examination, nasal endoscopy, etc.) and the subject has a positive response to any of the following temporary measures (based on patient history or office exam):   - Use of external nasal dilator strips (e.g., Breathe Right Strips) - Use of nasal dilator cones - Cottle Maneuver (manual lateral retraction of the cheek) or Modified Cottle Maneuver |  | Nasal valve is a primary or significant contributor to the subject's nasal obstruction as determined by the study investigator (based on clinical presentation, physical examination, nasal endoscopy, etc.), and the subject has a positive response to any of the following temporary measures (based on patient history or office exam):  • Use of external nasal dilator strips (e.g., Breathe Right Strips)  • Q-Tip test (manual intranasal lateralization)  • Use of nasal stents  • Cottle Maneuver (manual lateral retraction of the cheek) | Nasal valve is a primary or significant contributor to the subject's nasal obstruction as determined by the study investigator (based on clinical presentation, physical examination, nasal endoscopy, etc.) and the subject has a positive response to any of the following temporary measures (based on patient history or office exam):   - Use of external nasal dilator strips (e.g., Breathe Right Strips) - Q-Tip test (manual intranasal lateralization) - Use of nasal stents - Cottle Maneuver (manual lateral retraction of the cheek) |
|  | Willing and able to withhold anticoagulant medications during the perioperative period (3-day window on either side). |  |  |
| Willing and able to provide informed consent. | Willing and able to provide informed consent. | Willing and able to provide informed consent. | Willing and able to provide informed consent. |
| Willing and able to comply with the participant-specific requirements outlined in the study protocol. | Willing and able to comply with the participant-specific requirements outlined in the study protocol. | Willing and able to comply with the study protocol. | Willing and able to comply with the subject-specific requirements outlined in the study protocol. |
| Dissatisfaction with medical management as judged by the patient. Defined as failed medical therapy (eg, decongestants, antihistamines, and/or nasal sprays for an appropriate period of time), but a positive response to internal or external nasal dilators. |  |  |  |
| **Exclusion Criteria** | | | |
|  |  | Prior surgical treatment of the nasal valve. | Prior surgical treatment of the nasal valve |
| Rhinoplasty, septoplasty, inferior turbinate (IT) reduction, or functional endoscopic sinus surgery within the preceding 3 months. | Rhinoplasty, septoplasty, IT reduction, or other surgical nasal procedures within the preceding 6 months. | Rhinoplasty, septoplasty, IT reduction, or other surgical nasal procedures within the past 3 months. | Rhinoplasty, septoplasty, IT reduction or other surgical nasal procedures within the past twelve (12) months |
| Severe case of any of the following: septal deviation, turbinate hypertrophy, polyps, or ptotic nose tip believed to be the primary contributor to the participant’s nasal obstruction symptoms and warranting surgical intervention. | Severe case of any of the following: septal deviation, turbinate hypertrophy, polyps, or ptotic nose tip believed to be the primary contributor to the participant’s nasal obstruction symptoms and warranting surgical intervention. |  | Septal deviation, turbinate hypertrophy, polyps, or ptotic nose tip believed to be a significant contributor to the subject's nasal obstruction symptoms. |
| Any adjunctive surgical nasal procedure planned on the same day or within 6 months after the VivAer procedure. | Any adjunctive surgical nasal procedure planned on the same day or within 3 months after the VivAer procedure. | Anatomy that requires an adjunctive surgical nasal procedure on the same day or 3 months after the VivAer procedure. |  |
| Known or suspected allergies or contraindications for any general or local anesthetic agents. | Known or suspected allergies or contraindications for any general or local anesthetic agents. |  | Known or suspected allergies or contraindications for any general or local anesthetic agents and / or any antibiotic medications |
| Known or suspected to be pregnant or is lactating. | Known or suspected to be pregnant or is lactating |  | Known or suspected to be pregnant, or is lactating |
| Participating in another clinical research study. | Participating in another clinical research study. |  |  |
| Other medical conditions which in the opinion of the investigator would predispose the participant to poor wound healing or increased surgical risk, or poor compliance with the requirements of the study. | Other medical conditions which in the opinion of the investigator would predispose the participant to poor wound healing or increased surgical risk, or poor compliance with the requirements of the study. | Medical conditions which in the opinion of the treating physician would predispose the subject to poor wound healing or increased surgical risk. | Other medical conditions which in the opinion of the investigator could predispose the subject to poor wound healing or increased surgical risk |
|  | Known or suspected regular use of oxymetazoline (Afrin) nasal decongestant or oral steroids is exclusionary. |  |  |
|  | For sites participating in the CT substudy only: Active sinus condition (eg, significant sinus diseases, infection, or polyp formation) identified by CT. |  |  |
| Prior surgery of the lateral nasal wall, including cephalic resection of the lower lateral cartilage, dome division or suture plication, alar graft or spreader graft placement. |  |  |  |
|  |  |  | Chronic sinusitis, recurrent sinusitis, or allergies leading to nasal obstruction |

NOSE = Nasal Obstruction Symptom Evaluation; SSB = Septal Swell Body; IT = Inferior Turbinate; CT = Computed Tomography.

**Supplementary Table 2. Comparison of NOSE Q4 “Trouble Sleeping” Scores by OSA Status**

| **Visit** | **OSA+ n (%)** | **OSA+ Mean (SD)** | **OSA+ Change from Baseline (Mean Δ ± SD)** | **OSA+ Within-Arm p (value vs Baseline)** | **OSA− n (%)** | **OSA− Mean (SD)** | **OSA− Change from Baseline (Mean Δ ± SD)** | **OSA− Within-Arm (p-value) vs Baseline** | **Across-Arm**  **(p-value) OSA+ vs OSA−** |
| --- | --- | --- | --- | --- | --- | --- | --- | --- | --- |
| Baseline | 37 (10.7) | 3.1 (1.0) | – | – | 308 (89.3) | 2.9 (1.0) | – | – | 0.256 |
| 3 Months | 37 (10.9) | 1.8 (1.1) | −1.3 (1.1) | <0.001 | 303 (89.1) | 1.2 (1.2) | −1.7 (1.2) | <0.001 | 0.003 |
| 6 Months | 36 (11.0) | 1.3 (1.3) | −1.8 (1.3) | <0.001 | 292 (89.0) | 1.0 (1.1) | −1.9 (1.1) | <0.001 | 0.192 |
| 12 Months | 32 (10.8) | 1.3 (1.1) | −1.8 (1.1) | <0.001 | 260 (88.1) | 0.9 (1.1) | −2.0 (1.1) | <0.001 | 0.059 |
| 24 Months | 28 (10.9) | 1.3 (1.1) | −1.8 (1.1) | <0.001 | 227 (88.7) | 1.0 (1.2) | −1.9 (1.2) | <0.001 | 0.186 |

Comparison of Q4 Scores by OSA Status: Mean NOSE scores and NOSE “trouble sleeping” subcomponent are shown for participants with (OSA+) and without or unknown (OSA–) obstructive sleep apnea at each timepoint. Welch’s t‑test used for across‑arm comparisons; paired t‑tests for within‑arm pre‑ vs post comparisons. Negative Δ values indicate improvement.

**Supplementary Table 3. ESS Severity Progression and Comparison of Score Changes at 24 Months in VATRAC Cohort Patients with Baseline ESS ≥10 and ≥15**

| **ESS Severity Progression and Score Changes** | | | | | |
| --- | --- | --- | --- | --- | --- |
| **Measure** | **Baseline**  **(N = 106)** | **3 Months**  **(N = 106)** | **6 Months**  **(N = 101)** | **12 Months (N = 92)** | **24 Months (N = 73)** |
| **Severity Category** | | | | | |
| Unlikely Daytime Sleepiness (0–7) | 34 (32.1%) | 68 (64.2%) | 67 (66.3%) | 71 (77.2%) | 57 (79.2%) |
| Average Daytime Sleepiness (8–9) | 15 (14.2%) | 7 (6.6%) | 12 (11.9%) | 6 (6.5%) | 8 (11.1%) |
| Excessive Daytime Sleepiness (10–15) | 30 (28.3%) | 19 (17.9%) | 16 (15.8%) | 13 (14.1%) | 4 (5.6%) |
| Severe EDS (16–24) | 27 (25.5%) | 12 (11.3%) | 6 (5.9%) | 2 (2.2%) | 3 (4.2%) |
| **ESS Scores ≥10** | | | | | |
| n (%) | 53 (50%) | 53 (50%) | 51 (50.5%) | 46 (50%) | 34 (46.6%) |
| Mean (SD) | 15.7 (2.8) | 10.2 (5.5) | 8.6 (5.0) | 7.5 (4.3) | 6.6 (4.6) |
| Median | 16.0 | 11.0 | 8.0 | 7.0 | 6.0 |
| Min - Max | 11.0 – 22.0 | 0.0 – 20.0 | 0.0 – 20.0 | 0.0 – 18.0 | 0.0 – 19.0 |
| 95% CI | (14.9, 16.4) | (8.8, 11.7) | (7.3, 10.0) | (6.2, 8.7) | (5.1, 8.1) |
| LS Mean (95% CI) – Total Score (LMM-adjusted) | 15.64  (14.50,16.77) | 10.22  (9.09, 11.36) | 8.67  (7.52, 9.82) | 7.60  (6.41, 8.79) | 7.07  (5.76, 8.38) |
| **Change from Baseline** | | | | | |
| Change: Mean (SD) | 0.0 (0.0) | -5.4 (5.4) | -7.0 (5.0) | -8.1 (4.3) | -8.9 (4.6) |
| Change: Median | 0.0 | -4.0 | -7.0 | -8.0 | -10.0 |
| Change: Min - Max | 0.0 – 0.0 | -16.0 – 3.0 | -16.0 – 3.0 | -16.0 – 2.0 | -15.0 – 4.0 |
| Change: 95% CI | (0.0, 0.0) | (-6.9, -3.9) | (-8.4, -5.7) | (-9.4, -6.9) | (-10.5, -7.4) |
| p-value – CFB vs Baseline (LMM) | N/A | <0.001 | <0.001 | <0.001 | <0.001 |
| LS Mean (95% CI) (LMM–adjusted) | N/A | -5.39  (-6.69, -4.09) | -6.89  (-8.20, 5.58) | -7.93  (-9.26, -6.59) | -8.32  (-9.74, 6.90) |
| **% Change from Baseline** | | | | | |
| Mean (SD) | 0.0 (0.0) | -34.2 (34.5) | -44.7 (31.2) | -52.0 (26.4) | -57.6 (28.1) |
| Median | 0.0 | -26.3 | -50.0 | -56.4 | -62.0 |
| Min - Max | 0.0 – 0.0 | -100.0 – 21.4 | -100.0 – 25.0 | -100.0 – 15.4 | -100.0 – 28.6 |
| 95% CI | (0.0, 0.0) | (-43.5, -24.9) | (-53.3, -36.2) | (-59.6, -44.4) | (-67.1, -48.2) |
| **ESS Scores ≥15** | | | | | |
| n (%) | 27 (25.5%) | 27 (25.5%) | 26 (25.7%) | 23 (25%) | 17 (23.3%) |
| Mean (SD) | 18.0 (1.6) | 11.7 (5.4) | 9.9 (5.2) | 8.3 (4.5) | 7.6 (4.6) |
| Median | 18.0 | 13.0 | 9.5 | 7.0 | 7.0 |
| Min - Max | 16.0 – 22.0 | 3.0 – 20.0 | 2.0 – 20.0 | 4.0 – 18.0 | 2.0 – 19.0 |
| 95% CI | (17.4, 18.6) | (9.7, 13.7) | (7.9, 11.9) | (6.5, 10.2) | (5.4, 9.8) |
| LS Mean (95% CI) – Total Score (LMM-adjusted) | 18.03  (16.35, 19.70) | 11.73  (10.06, 13.40) | 9.98  (8.28, 11.67) | 8.50  (6.74, 10.27) | 8.05  (6.10, 10.00) |
| **Change from Baseline** | | | | | |
| Mean (SD) | 0.0 (0.0) | -6.3 (5.6) | -8.1 (5.2) | -9.8 (4.1) | -10.5 (4.4) |
| Median | 0.0 | -5.0 | -8.5 | -11.0 | -12.0 |
| Min - Max | 0.0 – 0.0 | -16.0 – 3.0 | -16.0 – 2.0 | -16.0 – 0.0 | -15.0 – -1.0 |
| 95% CI | (0.0, 0.0) | (-8.4, -4.2) | (-10.1, -6.1) | (-11.5, -8.2) | (-12.6, -8.4) |
| p-value – CFB vs Baseline (LMM) | N/A | <0.001 | <0.001 | <0.001 | <0.001 |
| LS Mean (95% CI) (LMM-adjusted) | N/A | -6.32  (-8.26, -4.38) | -8.04  (-10.00, -6.08) | -9.35  (-11.36,-7.34) | -9.68  (-11.82, -7.53) |
| **% Change from Baseline** | | | | | |
| Mean (SD) | 0.0 (0.0) | -34.5 (31.1) | -44.8 (29.3) | -54.8 (23.1) | -58.5 (24.4) |
| Median | 0.0 | -26.3 | -48.5 | -63.2 | -63.6 |
| Min - Max | 0.0 – 0.0 | -82.4 – 17.6 | -88.9 – 12.5 | -77.8 – 0.0 | -88.2 – -5.0 |
| 95% CI | (0.0, 0.0) | (-46.2, -22.7) | (-56.0, -33.5) | (-64.2, -45.3) | (-70.1, -46.9) |
| **Pairwise Comparisons of ESS Changes Using a Linear Mixed Model** | | | | | |

| **Visit Comparison** | **Estimate (Δ ESS)** | **95% CI** | **p-value** |
| --- | --- | --- | --- |
| 3 Months - 6 Months | 0.90 | (0.1, 1.7) | 0.014 |
| 3 Months - 12 Months | 1.67 | (0.9, 2.5) | <0.001 |
| 3 Months - 24 Months | 1.64 | (0.8, 2.5) | <0.001 |
| 6 Months - 12 Months | 0.77 | (0.0, 1.6) | 0.066 |
| 6 Months - 24 Months | 0.74 | (-0.1, 1.6) | 0.128 |
| 12 Months - 24 Months | -0.03 | (-0.9, 0.9) | 1.000 |

Categorical changes in Epworth Sleepiness Scale (ESS) severity over time among VATRAC participants (N = 106 at baseline) are tabulated. Categories include established clinical cutoffs for daytime sleepiness. Values are n (%). ESS scores are summarized for participants with baseline ESS ≥10 and ESS ≥15. Data include raw and adjusted means, change from baseline, and percent change from baseline through 24 months.

Longitudinal analyses were conducted using linear mixed-effects models (LMMs) with Visit as a fixed effect, baseline ESS score as a covariate, and Subject ID as a random intercept. LS means and 95% confidence intervals are derived from the LMM, with Dunnett–Hsu multiplicity adjustment for comparisons versus baseline. Percent changes are descriptive; statistical testing was performed using the LMM. Pairwise comparisons of adjusted ESS scores between follow-up visits were also conducted using the LMM framework. Estimates reflect differences in adjusted LS mean ESS scores at each interval (3–6 months, 3–12 months, etc.).

Abbreviations: ESS = Epworth Sleepiness Scale; EDS = excessive daytime sleepiness; N = number of subjects; n = number of observations; SD = standard deviation; CI = confidence interval; LS = least squares; LMM = linear mixed-effects model; CFB = change from baseline; %CFB = percent change from baseline.
